# EuleApp©: a computerized adaptive assessment tool for early literacy skills

**DOI:** 10.3389/fpsyg.2025.1522740

**Published:** 2025-04-25

**Authors:** Melike Yumus, Christina Stuhr, Marlene Meindl, Haug Leuschner, Tanja Jungmann

**Affiliations:** ^1^Department of Special Needs Education and Rehabilitation, Carl von Ossietzky University of Oldenburg, Oldenburg, Germany; ^2^Faculty of Philosophy, Institute for Sports Science, University of Rostock, Rostock, Germany; ^3^Department of Special Education and Rehabilitation, University of Rostock, Rostock, Germany; ^4^DHL Data Science Seminare GmbH, Köln, Germany

**Keywords:** early literacy, digital assessment, preschool age, item response theory, computerized adaptive test, psychometric validation

## Abstract

**Introduction:**

Ample evidence indicates that assessing children’s early literacy skills is crucial for later academic success. This assessment enables the provision of necessary support and materials while engaging them in the culture of print and books before school entry. However, relatively few assessment tools are available to identify early literacy skills, such as concepts of print, print awareness, phonological awareness, word awareness, alphabet knowledge, and early reading. The digital landscape presents new opportunities to enhance these assessments and provide enriching early literacy experiences. This study examines the psychometric properties of an adaptive assessment tool, EuLeApp©, focusing on its reliability and concurrent validity.

**Methods:**

Data involved 307 German kindergarten children (M_age_ = 64 months old, range = 45–91). A Computerized Adaptive Testing (CAT) method, grounded in Item Response Theory (IRT), was employed to develop an adaptive digital tool for assessing early literacy competencies. We utilized an automatic item selection procedure based on item difficulty and discrimination parameters for the 183-item pool to ensure a precise and efficient assessment tailored to each child’s ability level.

**Results:**

The 4-parameter Logistic (4PL) model was identified as the best-fitting model for adaptive assessment, providing the highest precision in estimating children’s abilities within this framework.

**Discussions:**

The findings support the idea that the adaptive digital-based assessment tool EuLeApp© can be used to assess early literacy skills. It also provides a foundation for offering individualized and adaptable learning opportunities embedded in daily routines in daycare centers.

## 1 Introduction

Many studies have highlighted significant differences in reading and writing outcomes between strong and weak readers from early years through high school (Buckingham et al., 2013; McArthur et al., 2020). For instance, in Germany, recent investigations reveal that almost two in five fourth graders score “below basic” in their reading and writing skills, indicating they struggle to read and understand simple texts (European Commission, 2023; McElvany et al., 2023). This finding underscores a broader issue in educational systems worldwide: the failure to identify children at risk of reading difficulties early enough to provide timely and adequate interventions (Adams, 1994; Catts et al., 2001; Justice and Ezell, 2001). This disparity also ties into the dyslexia paradox, where the most effective interventions occur before a child experiences academic failure (Shaywitz, 1998). Addressing this issue requires reliable, efficient, and adaptive diagnostic tools capable of predicting early literacy skills from preschool onward. Such tools would enable educators to detect literacy difficulties early, providing the foundation for timely and targeted interventions (Care et al., 2018; Gonski et al., 2018). This approach also aligns with ecological and sociocultural approaches to assessment, which emphasize a child’s “readiness to learn” and the zone of proximal development, where scaffolding by teachers supports learning from the familiar to the unfamiliar (Vygotsky, 1978). Through these approaches, assessment becomes a means to monitor a child’s progress over time, fostering an adaptive learning environment. The app’s computerized adaptive testing (CAT) framework dynamically adjusts to each child’s ability level, making it possible to identify literacy difficulties, including dyslexia, early on. This approach is critical for reducing the gap between strong and weak readers by tailoring assessments and providing opportunities for interventions to each child’s unique needs, thus preventing the long-term consequences of untreated literacy deficits.

The accurate and timely assessment of early literacy skills is essential for early identification of learning gaps, customizing daily literacy activities, monitoring progress, and making data-driven adjustments to narrow achievement disparities. Thus, this project aimed to develop an adaptive assessment tool and to demonstrate the reliability and concurrent validity of EuLeApp© in a German sample of children aged 4–7 years. The adaptive approach is essential because it allows for precise performance estimates by adjusting the difficulty and number of items based on each child’s ability level (Weiss, 2004). By leveraging CAT, EuLeApp© maximizes both efficiency and accuracy, making it a valuable tool for early literacy assessment. Technology-based solutions, such as the EuLeApp©, hold great potential in this area.

### 1.1 The importance of individualized assessment of early literacy skills

Early literacy encompasses a range of skills related to oral language, phonological awareness, word awareness, print awareness, concepts of print, alphabet knowledge, and narrative abilities that develop before children formally learn to read and write. Phonological awareness refers to the ability to detect the smallest sound units within words. When children develop this skill, they understand that language can be analyzed and manipulated, a key milestone in their literacy journey toward understanding the concept of words (Gee, 2012; Snow and Dickinson, 1991). Word awareness involves recognizing that words, as elements of language, have properties independent of their meaning. For example, children learn to connect printed words in their oral vocabulary while learning to read. This awareness also includes understanding word boundaries and what constitutes a word (Justice and Ezell, 2001). The term print awareness refers to the knowledge that print carries meaning, and differs structurally from other sign systems (e.g., numbers). To develop print awareness, it is crucial for young children to be exposed to letters and written text in their environment. Familiarity with books and print culture also involves understanding the characteristics of books and how they are read, which relates to concepts of print (Meindl and Jungmann, 2019; Piasta et al., 2012). Alphabet knowledge entails recognizing the characteristics of different graphemes and associating them with their corresponding phonemes. Early reading skills include understanding that words are made up of graphemes (Elimelech and Aram, 2020; Morrow, 2007). Narrative skills reflect children’s ability to produce a fictional or real account of a temporally sequenced experience or event (Engel, 1995). All these aspects represent crucial milestones in the successful development of reading and writing (Justice and Ezell, 2001).

Early literacy tasks such as phonological awareness, print awareness, and word awareness reflect how children process and integrate the relationships between various linguistic rule systems on the metacognitive level. Nagy and Anderson (1995) documented that metalinguistic awareness helps young children to become aware of the structure of their writing system and its relationship to their spoken language. For instance, children with higher metalinguistic awareness perform better on tasks related to concepts of print and phonological awareness, both of which are strong predictors of reading success (Chaney, 1994). Without proper assessments, children in need of additional support may be overlooked, leading to long-term consequences such as ongoing academic difficulties, low self-esteem, and limited future opportunities (Brassard and Boehm, 2007; Justice et al., 2002; OECD, 2013). Screening early literacy skills with standardized assessments has gained significant attention in early childhood for various reasons: (a) to evaluate a child’s strengths and weaknesses in specific areas, (b) to identify key target skills and provide tailored support, (c) to structure educational programs, (d) to monitor children’s progress over time, and (e) to improve educational outcomes by facilitating a smoother transition to school. Assessment tools also offer significant benefits at different stages of assessment, from testing to linking appropriate interventions, by collaborating with children’s parents and other stakeholders to help overcome disadvantages. Despite the benefits of standardized testing, research shows that data-driven decision-making remains significantly underutilized in early education. Children enter kindergarten with a wide range of literacy and language abilities (Catts et al., 2002), making individualized feedback essential (Brassard and Boehm, 2007; McLachlan et al., 2018; Snow and van Hemel, 2008). To address these needs, computerized adaptive testing (CAT) provides an innovative solution for assessing children’s literacy skills. By dynamically adjusting to each child’s ability level, CAT improves measurement precision while reducing the required test items. As a cutting-edge tool, EuLeApp© leverages CAT to streamline the assessment process, ensuring both accuracy and efficiency. This adaptability allows practitioners to deliver individualized feedback, enabling more targeted interventions and improving educational outcomes.

### 1.2 The importance of innovative assessment tools

Given the ongoing expansion of digital media in educational settings (OECD, 2015) and the demand for more effective assessment methods, researchers have increasingly focused on the potential of digital tools for enhancing educational processes. These tools offer greater efficiency (Marsh et al., 2020) and provide visually engaging reports for tracking learning progress (Neumann et al., 2019). The use of digital assessment tools is increasing for numerous reasons, such as technological portability and ease, the touchscreen’s tremendous potential to reach young children, and the need for mobile, adaptable, and accessible assessment tools (Neumann et al., 2019). Furthermore, online assessment tools have become widespread and are actively used. For example, Ho et al. (2024) developed a short-term online test to assess word reading. This test, which covers a wide age range, is primarily designed for individuals who can read to some degree at a basic level (ages 7 and above). Tablet-based assessments, on the other hand, allow for greater control over the evaluation process for younger children and may be a more suitable alternative. Additionally, app-based assessments offer the advantage of functioning offline, making them more accessible in diverse settings such as homes, preschools, and clinics, where stable internet access is not always available.

App-based assessments can be administered anywhere with a suitable device, making it easier for educators and researchers to assess children in various settings, including homes, classrooms, and clinics. Another benefit of mobile media devices is that they can help advance the goal of reaching many children for educational opportunities and equity because of their low costs and good accessibility (Hirsh-Pasek et al., 2015). Additionally, traditional paper-pencil assessments usually require considerable time, effort, and expertise, such as organizing, rewriting, and preparing children for the test in person (Schildkamp and Kuiper, 2010). Also, some assessment procedures rely on contextual factors such as observation, making it challenging to maintain strict objectivity (Hindman et al., 2020). Moreover, children may not be able to show their best performance when assessed by someone they do not know (Halliday et al., 2018). In contrast, computerized assessments offer the potential to gain insights into children’s responses, such as disengagement, rapid guessing, or unexpected answers (Bulut and Cormier, 2018; Lee and Jia, 2014; Wise and Kong, 2005). Thus, app-based assessments enable teachers to receive prompt feedback, facilitating more effective support and remediation. However, technology-based assessments also raise concerns regarding developmental appropriateness, item development, psychometric validity, and teacher training (Bulut and Cormier, 2018; Neumann et al., 2019). Tablet-based assessments are common in many schools across the United States, where they are used to assess and monitor students’ performance in mathematics, reading, and science throughout the school year (Davey, 2005; Sharkey and Murnane, 2006). Despite the increasing integration of technology in education, the development of digital tools specifically for early literacy assessment remains limited, underscoring the need for further innovation. Emerging research shows that only a few app-based tools are available to assess children’s language and literacy skills in the early years. One such tool is Logometro^®^, a reliable app-based test that evaluates children’s phonological awareness, listening comprehension, vocabulary, narrative skills, speech, morphological awareness, and pragmatic skills (Antoniou et al., 2022). Administered through a specially developed Android app, Logometro^®^ allows for accurate directional vocalization and easy capture of children’s responses via touchscreens and direct recordings (Antoniou et al., 2022). Another innovative app, QUILS (Golinkoff et al., 2017) focuses on assessing children’s language-learning processes, offering insights into how they acquire new words and grammatical structures. Golinkoff et al. (2017) demonstrated that a technology-based assessment tool measuring children’s phonological awareness and letter knowledge was efficient in terms of time usage and effectively differentiated these skills. One app was also developed by Neumann (2018) to assess children’s letter knowledge and vocabulary using both expressive and receptive response formats. The valuable insights gained from such innovative assessment tools can make them more attractive, accurate, and accessible for educators and parents.

### 1.3 Computerized adaptive testing (CAT)

Computerized Adaptive Tests (CAT) dynamically adjust to a child’s ability by selecting test items based on their responses. Unlike traditional fixed-form tests, CAT tailors the difficulty of each item to match the child’s performance, selecting more challenging questions after correct answers and easier ones after incorrect responses (Meijer and Nering, 1999; Weiss and Kingsbury, 1984). This individualized approach helps maintain engagement and ensure a more accurate assessment of abilities (Tomasik et al., 2018; Wise, 2014). CAT relies on an Item Response Theory (IRT) calibrated item bank, selecting items sequentially to estimate a child’s ability (*θ*) more precisely with fewer items than conventional tests (Chalmers, 2015; Keuning and Verhoeven, 2008). The efficiency of CAT is enhanced by large item banks, allowing the test to adapt to individual performance effectively (Nelson et al., 2017). IRT provides a robust framework for interpreting test scores, as it allows for accurate item selection and predicts the likelihood that a child will respond correctly based on their skill level (Bjorner et al., 2007; Baker, 2001). One key advantage of IRT is that item parameters remain stable across different samples, meaning they are not dependent on the specific group being tested (Magis and Barrada, 2017). Additionally, IRT offers a precision measure or standard error for each skill estimate (He and Min, 2017), providing insight into the accuracy of the assessment across varying levels of ability (Weiss, 1982).

### 1.4 Present study

The quality of assessment tools is important for educators to understand what children are expected to learn before formal school readiness. A primary aim of the current study is to adapt a digital assessment tool from the paper-pencil-based EuLe 4–5 assessment (Meindl and Jungmann, 2019), a standardized tool designed to assess narrative and early literacy skills in German children aged 4;0 to 5;11 years. Based on this goal, the study also aims to validate the EuLeApp© as a digital, adaptive assessment tool for children aged between 4;0 and 7;11 years. For this, a Multidimensional Computerized Adaptive Test (MCAT) was used based on Item Response Theory (IRT), allowing for individualized and precise measurement of children’s early literacy skills across multiple dimensions. An item pool was constructed through calibration based on the content of the items, and model fit was estimated and established using an IRT model.

These research questions will be addressed as follows:

1. Does the EuLeApp© screening tool accurately assess early literacy skills in children aged 4 to 7 years?
2. How can item response theory (IRT) be used to optimize item difficulty in computerized adaptive testing (CAT) for assessing children’s early literacy skills?

## 2 Materials and method

### 2.1 Sample

The sample consisted of *N* = 307 kindergarten children (M_age_ = 64 months, range = 45–91) before entering formal schooling in Mecklenburg-West Pomerania and Lower Saxony in Germany. The sample distribution of boys (*n* = 170, 55.4%) and girls (*n* = 137, 44.6%) was approximately equal. In terms of the distribution of children’s ages, 31.3% were 4 years old, 47.2% were 5 years old, 13.0% were 6 years old, and 8.5% were 7 years old. Data were primarily collected from kindergartens in middle- and high-socioeconomic regions. All parents were informed about the study, and written consent was obtained. Table 1 provides an overview of the participant demographics, including age distribution, gender, and other key characteristics.

**Table 1:** Participant demographics (*N* = 307).

Variable	Category	*N*	Frequency (%)
State	Mecklenburg-West Pomerania	171	55.7
	Lower Saxony	136	44.3
Gender	Boys	170	55.4
	Girls	137	44.6
Age group	4 years	96	31.3
	5 years	145	47.2
	6 years	40	13.0
	7 years	26	8.5
Parental education	Secondary education	123	40.1
	Vocational training	53	17.3
	Higher education	131	42.6
Linguistic diversity	Monolingual (German)	253	82.4
	Bilingual	24	7.8
	Missing data	30	9.8

### 2.2 Measures

#### 2.2.1 Early literacy assessment app (EuLeApp©)

**Figure 1:**
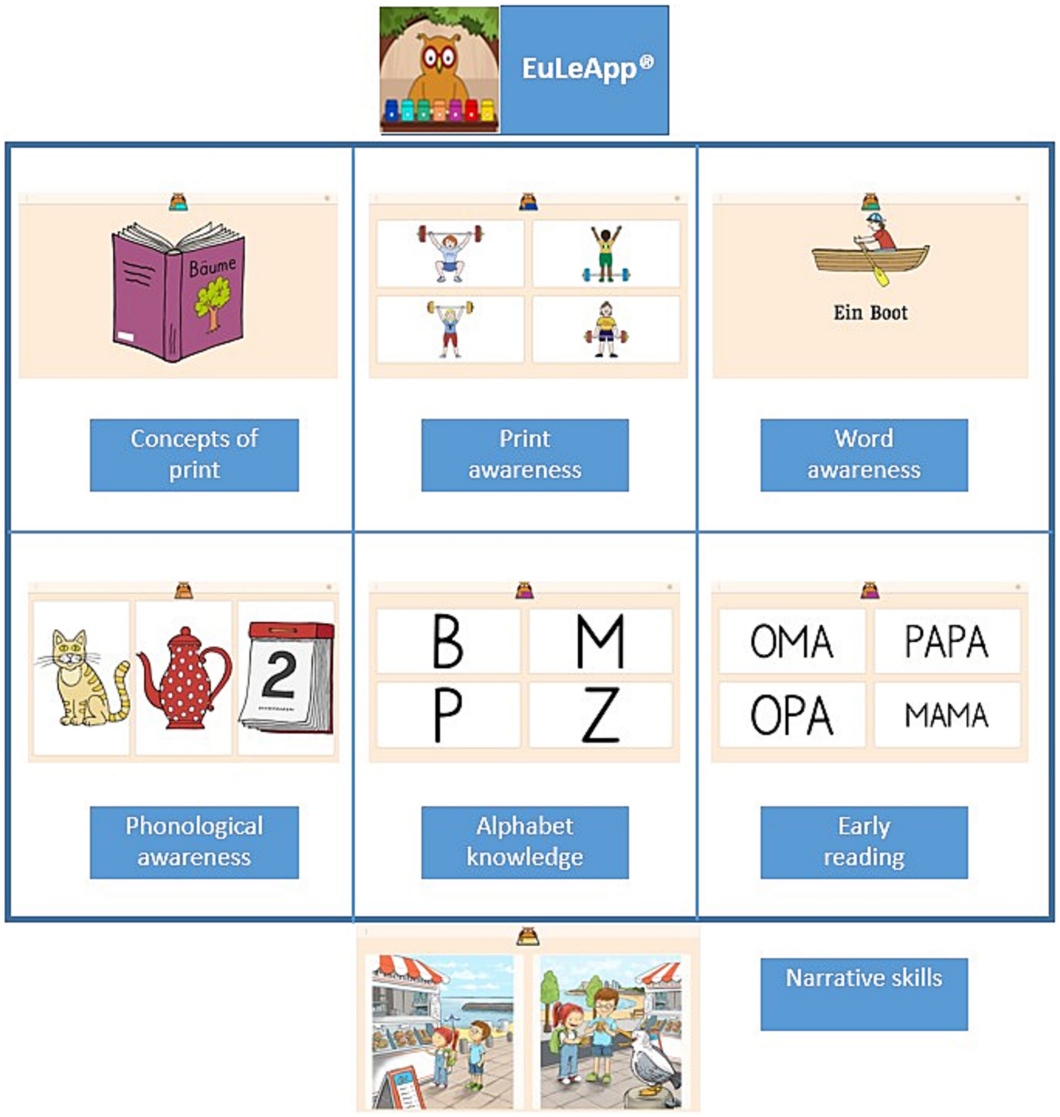
EuLeApp© subtests with sample test items.

To assess early literacy between the ages of 4 and 7 years, we administered the EuLeApp©, a digital multiple-choice test developed to measure key early literacy content areas, including the following: (a) the concepts of print, (b) print awareness, (c) word awareness, (d) phonological awareness, (e) alphabet knowledge, and (f) early reading (g) narrative skills (Figure 1).

#### 2.2.2 EuLeApp© features

During the development of the EuLeApp© prototype, four key design features were incorporated to support the self-assessment process: definition, modeling, practice, and motivation (Carson et al., 2015). The “definition” feature provides a brief explanation of what the child will do and how the task works. While the “modeling” feature demonstrates how to use the system and what is expected of the child (e.g., how to interact with the tool and respond to questions), the “practice” feature allows the child to practice how the system works before the actual assessment. The goal of the “motivation” feature is to motivate and encourage children to keep them engaged in completing the test.

This digital assessment tool can be completed in a single session (∼20 min). It requires no formal training and can be automatically administered and scored using its software program, except for the picture story for the narrative part “Seagull Marius.” For only this narrative part, the pedagogical professionals have to analyze the realized macrostructure of the child’s narration with the help of a protocol sheet.

The EuLeApp©’s subscales, each tapping into different and overlapping skills, were developed to provide teachers, pedagogic staff, researchers, and parents with sufficient information to interpret children’s early literacy performance. The assessment follows a multiple-choice format, which is widely recognized as an effective method for direct measurement (Haladyna, 2013; Raymond et al., 2019). Accordingly, each child is shown with a picture or figure with four response options on the screen (one target response, three distractors). Within these six subscales (Figure 1), we presented children with 175 items in total. Item indicators are equivalent to items in a conventional test. Item selection considered linguistic (e.g., bilingual, multilingual), socioeconomic, and cultural diversity (Levine et al., 2020). In this context, we used short and clear instructions. Besides being more effective than open-ended questions, another important reason for structuring the test in a multiple-choice format is considering equity for children from different backgrounds (Bruder, 1993; Neumann et al., 2019).

*Concepts of print*: This task comprises 40 items, and children need to identify print-related tasks such as reading directions from left to right or where to start reading (Cronbach’s *α* = 0.93). Based on the questions, children are required to tap on the correct part of the screen.

*Print awareness*: The assessment of print awareness comprises a 19-item subtest (Cronbach’s *α* = 0.76). The task of the children is to distinguish between words, icons, and symbols (“Tap on the letter”; “Tap on writing”). Each child is presented with four pictures, one of which is the target and the other three serving as distractors, and is expected to tap either on the word or the letter corresponding to the target.

*Words awareness*: This task consists of 11 items that increase in difficulty gradually (Cronbach’s α = 0.79). During this assessment, children encounter short texts and are directed to tap on specific elements, such as the first, second, or space between two words (“Tap on the space between two words”).

*Phonological awareness*: The phonological awareness subtest comprises 29 items (Cronbach’s α = 0.72) and assesses the ability of two key components: synthesizing syllables and phonemes to form words and analyzing words’ syllabic and phonemic structure. For example, tasks include identifying the initial sounds of words (e.g., “Here you can see three pictures: Grandma, Mum, Apple. Touch the picture that starts with /m/”).

*Alphabet knowledge*: This subtest consists of 45 items (Cronbach’s α = 0.89). To evaluate children’s alphabet knowledge, they are presented with phonetic realizations of letters and are asked to select the corresponding letter from a set of four options (e.g., “Tap the /m/”).

*Early reading*: In the early reading segment, children are asked to name the 36 letters of the alphabet (Cronbach’s α = 0.96). Items such as “Tap on /am/,” “Tap on /mama/” (receptive segment), and “What is written here?” (productive segment) assess children’s first receptive and productive reading abilities on the syllable and word level.

Table 2 provides descriptive statistics, reliability estimates, and distribution metrics (skewness and kurtosis) for each subscale. While Cronbach’s α values indicate good internal consistency across the EuLeApp© scales, particularly for Concepts of Print, Alphabet Knowledge, and Early Reading, the inter-item correlation values were comparatively lower. Additionally, skewness and kurtosis values indicate that most subscales exhibit approximately normal distributions, with no extreme deviations from normality, supporting the reliability and usability of the scales.

**Table 2:** Descriptive statistics and internal consistency of the scores in each subscale (*N* = 307).

Scale	*M*	*SD*	*M* IIC	*SD* IIC	Cronbach’s α	Skewness	Kurtosis
Concept of prints	23.7	10.4	0.277	0.156	0.939	−0.065	−0.891
Print awareness	11.8	3.7	0.157	0.117	0.769	−0.437	−0.058
Word awareness	5.3	3.2	0.244	0.106	0.796	0.294	−0.678
Phon. Awareness	18.9	4.3	0.082	0.083	0.729	0.821	0.373
Alphabet knowledge	19.3	9.0	0.152	0.083	0.892	0.782	0.274
Early reading	6.9	8.9	0.427	0.113	0.962	0.14	−0.372

*M IIC, Mean of Inter-Item Correlation; SD IIC, Standard Deviation of Inter-Item Correlation.*

In addition, the EuLeApp© includes a narrative measure in which children retell the story of Seagull Marius after viewing seven pictures. Therefore, this section of the assessment follows a different methodological approach, and its reliability evaluation is still ongoing.

The EuLeApp© was designed with the understanding that children can comfortably engage with tablets. The test begins with a short, child-friendly explanation and an example of how they will conduct it, and it provides a short practice. After an item and its audio are presented, children respond to the question by touching one of the options on the screen. The test then continues with children viewing items one by one. The software is configured so that children can respond flexibly.

The test automatically moves through subtests, and children view short, fun, animated scenes throughout the assessment as a break from the tasks. According to Campbell and Jane (2012), motivation is considered one way to stimulate children’s engagement. Therefore, children are regularly given positive feedback (e.g., “Well done!” or “Excellent!”) and visual, colorful gift boxes after each subtest.

##### 2.2.2.1 Scoring and coding

To describe the child’s strengths and weaknesses, a specialized database architecture was used to record the child’s interactions with each task on the tablet. An automated scoring system provides real-time feedback by evaluating whether each response is correct or incorrect. Most indicators are binary, designed to detect the presence or absence of a correct answer for each item. By automating this process, the system can quickly and accurately generate feedback highlighting the child’s strengths and identifying areas needing improvement. In other words, each early literacy domain is scored across a different number of items, and assessments of the children’s answers, false (0) or true (1) scores, are determined by their success or failure on the task. The coded items are considered the primary data source for the scoring process. Evaluation indicators are classified based on the child’s answers for each scale. Each assessment includes the child’s total response time, item counts, and each child is coded with a unique ID code. To capture the required data, once the indicators are determined for items in every subscale, difficulty differences based on these item indicators are (automatically) determined.

Children’s performance is reported as a score for each scale, allowing researchers and practitioners to monitor children’s progress. Furthermore, to facilitate the classification of early literacy skill levels, the assessment results in a color-coded ranking list with “traffic light” analogy (Templin and Henson, 2010). Children are categorized as “red” (at risk), “orange” (monitoring needed), or “green” (on track) based on their performance in key components of early literacy. This feature provides a viable way to identify children needing additional support, reinforcing the tool’s ability to differentiate between skill levels and guide targeted interventions. The reports are also displayed on the children’s profile pages and include short demographic information such as the child’s age, gender, and kindergarten/school. The App’s user-friendly interface allows for straightforward administration and scoring, making it a valuable resource for educators and researchers in early childhood education.

#### 2.2.3 Language competence

Children’s language competence was assessed using standardized German language tests, selected based on age: (a) “Language level test for children aged 3–5 years” (Sprachentwicklungstest für Kinder 3–5 [SET 3–5]; Petermann et al., 2016), (b) “Language level test for children aged 5–10 years” (Sprachentwicklungstest für Kinder 5–10 [SET 5–10]; Petermann, 2018). The SET 3–5 consists of 12 subtests that measure a child’s receptive language processing skills (understanding, recording), productive language processing skills (own speech acts), and auditory memory skills (language memory). The internal consistency for administered subtests from the SET 3–5 ranged between *α* = 0.70 and α = 0.93 (Petermann et al., 2016). The SET 5–10 consists of 8 subtests to measure a child’s vocabulary, semantic relations, processing speed, language comprehension, language production, grammar/ morphology, and auditory memory. The internal consistency for administered subtests ranges between α = 0.71 and α = 0.91 for the SET 5–10 (Petermann, 2018).

### 2.3 Procedures

Prior to the start of the study, university ethics approval was received, and permission to assess children was obtained from the head educators of a total of 15 kindergartens. Before administering the EuLeApp©, children’s language skills were evaluated to ensure that they possessed sufficient language comprehension. This step was essential in preventing the misinterpretation of literacy test results due to underlying language receptive problems and ensuring that literacy performance was accurately measured. Then, we assessed early literacy skills in the daycare centers using the prototype of the EuLeApp© on a tablet. The test practitioners were master’s students, PhD candidates, and postdoctoral fellows, all of whom completed two training sessions: one on understanding the assessment tool and its usage, and another on practical test implementation. All assessments were conducted individually. Before the test began, practitioners informed the children about the goal of the test and how it would help them, reassuring them that the test would not show everything they knew and could do and that they had plenty of time to answer the questions. Sitting beside the child, the test practitioners asked the child to practice tapping on the screen before beginning the assessment. Once the evaluation began, the practitioners did not answer the children’s questions or provide any tips to ensure standardized administration. During the EuleApp© assessment procedure, standard administration and scoring procedures were followed.

### 2.4 Data analysis strategy

**Figure 2:**
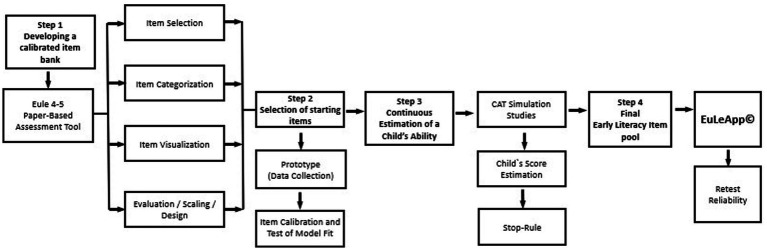
The development of an adaptive app-based early literacy assessment tool.

Figure 2 outlines the structured process used for the CAT analysis in EuLeApp©: (a) Developing a calibrated item bank: Relevant items from the EuLe 4–5 paper-based assessment tool were selected, categorized, and visualized to ensure consistency between the paper and digital formats. (b) Selection of starting items: A prototype was developed, and data were collected for item calibration, with model fit tested for accuracy. (c) Continuous estimation of a child’s ability: A child’s ability was continuously estimated during CAT simulation studies, applying a stop rule based on predefined precision criteria. (d) A final item pool was established based on simulations, integrated into EuLeApp©, and validated through reliable retest processes.

The R package mirtCAT was used for psychometric development of the app’s multidimensional computerized adaptive test (MCAT) based on Item Response Theory (IRT). Initially, items were adapted from the EuLe 4–5 test, whose content and construct validity had been previously established (Meindl and Jungmann, 2019). A digital platform was then created to deliver the assessment, incorporating multimedia features (vocal instructions, touch interactions, and graphics) to enhance children’s engagement and navigation. In the next step, data collection marked the first calibration phase of item pool development. A series of confirmatory IRT models were used to estimate item parameters and assess the effectiveness of the MCAT in providing individualized early literacy assessments. Both exploratory and confirmatory Item Factor Analysis (IFA) were conducted to validate the item structure, removing items misaligned with the identified factors to improve accuracy and reliability. Fit indices, including the Comparative Fit Index (CFI), Tucker–Lewis Index (TLI), Root Mean Square Error of Approximation (RMSEA), and Standardized Root Mean Square Residual (SRMR), were applied to evaluate the calibration data against the proposed six-factor model (Brown, 2015).

To address potential estimation challenges and improve model convergence, the model was divided to reduce the number of estimated parameters: the first submodel included scales 1–4, while the second submodel included scales 5–6. Using these submodels, exploratory and confirmatory IFA models were developed. Structural analysis indicated that items 9–40 exhibited a bifactor structure (Chen et al., 2006; Dunn and McCray, 2020). This meant that the “Concepts of Print” scale could only be derived if the model incorporated two additional dimensions related to the item-specific use of numbers and images, which did not correlate with other dimensions. These were defined as “Numerical Writing Awareness” and “Iconic Writing Awareness.” Items 1–8 were also deemed usable when assigned to new dimensions, further strengthening the bifactor structure of this scale. Based on these findings, the structural analysis was refined using a model with six dimensions for the EuLeApp© scales and two item-specific dimensions for numbers and images. Six items were removed due to poor model fit, as they could not be assigned to any specific dimension.

**Figure 3:**
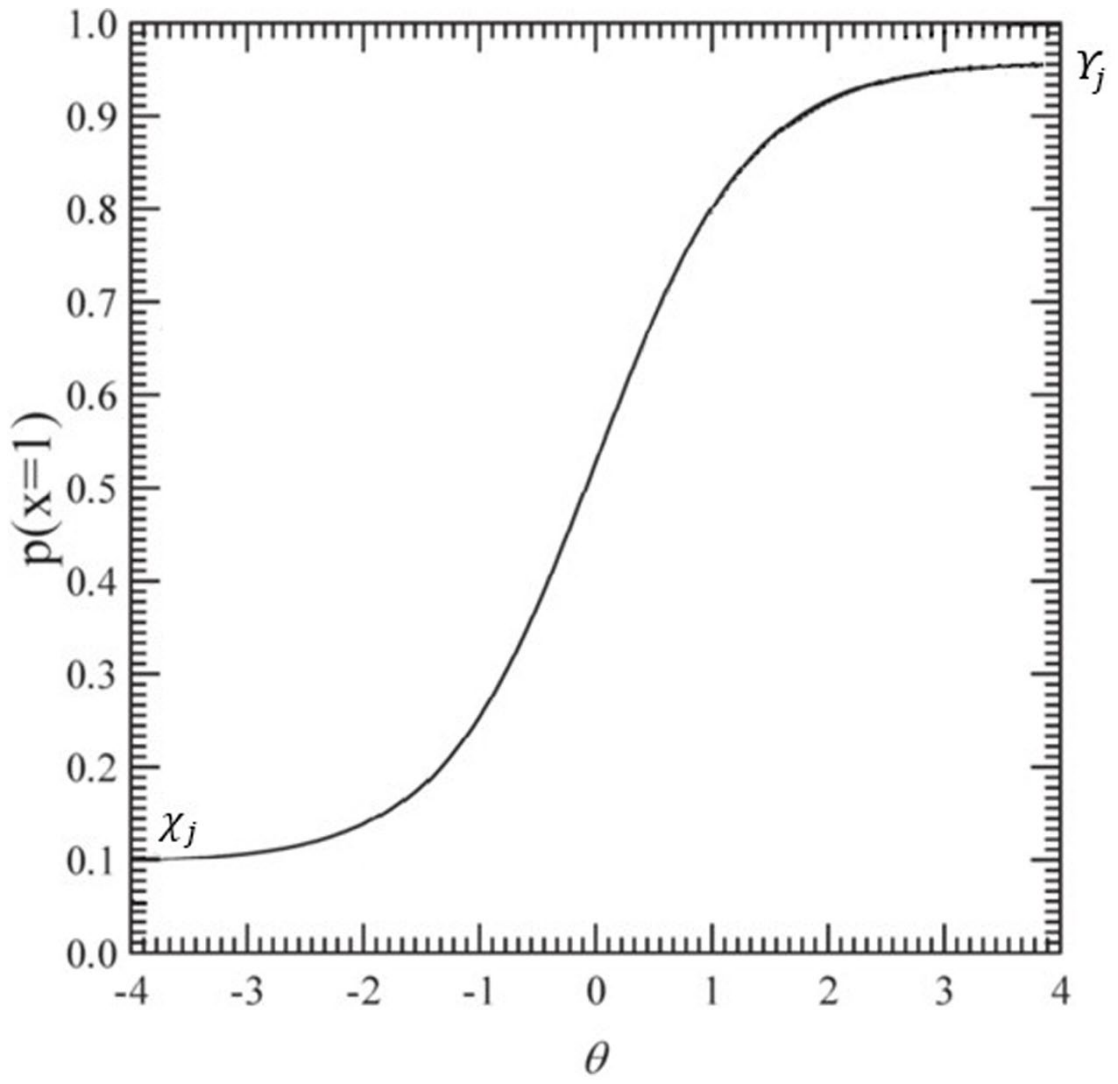
A typical item characteristic curve for the 4PL IRT model. P(*θ*) represents the probability of a correct response given the ability level. $\chi_{j}$ (ability). This represents the ability level of the individual, typically ranging from very low to very high values. ${ϒ}_{j}$ (probability of correct response). This represents the probability of answering the item correctly, ranging from 0 to 1. 4PL = four-parameter logistic.

As shown in Figure 3, the IRT curve of the 4-Parameter Logistic (4PL) model is compressed in the y-direction, ensuring that it remains within the probability range defined by the lower bound $\chi_{j}$ and the upper bound ${ϒ}_{j}$ (Yen et al., 2012). This structure sets minimum ( $\chi_{j}$ ) and maximum $\left({ϒ}_{j}\right)$ probabilities for the correct response to each item j , independent of the test-taker’s ability level. This allows the model to account for guessing (lower asymptote) and disengagement (upper asymptote), providing a more accurate representation of performance.

While the lower asymptote captures the probability of randomly guessing an item correctly, it is also crucial to consider factors that might cause young children to respond incorrectly to easy items despite possessing the required knowledge. Issues such as inattention, attention deficits, socially desirable responding, external distractions, or a lack of motivation to engage with simple tasks may contribute to this variability (Liao et al., 2012). To better accommodate these influences, the 4PL model incorporates an upper asymptote, which accounts for the possibility that even highly skilled individuals may not always respond correctly due to carelessness, disengagement, or momentary lapses in concentration (Antoniou et al., 2022; Barton and Lord, 1981).

From a developmental perspective, the 4PL model provides a more precise estimate of young children’s abilities. By accounting for cognitive and behavioral fluctuations common in early development, this model offers improved sensitivity to response patterns that may be influenced by variability in attention, motivation, and task engagement (Anderson et al., 2002). These features make the 4PL model particularly useful in assessing young learners, where performance is not solely determined by ability but also by contextual and developmental factors.

Specific item selection criteria and stopping rules were defined for the item bank used in the Computerized Adaptive Test (CAT) analysis process to enhance testing efficiency and precision (Ebenbeck and Gebhardt, 2022). Standard techniques, including the selection of starting items, regression analysis, and stopping rules, were implemented to optimize these goals (Roberts et al., 2000). In EuLeApp©, item difficulty is adjusted based on children’s responses. To identify age-appropriate starting items, 24 of the 183 available items were selected based on content considerations, ensuring that the MCAT process began with items that were neither too easy nor too difficult for each age group. A regression analysis was then conducted to analyze the influence of these starting items on the standard error of measurement for determining personal abilities. The stopping rule was set based on test precision, where the CAT algorithm assesses whether the confidence interval falls within specified limits. When this criterion is met, the algorithm concludes the assessment for that construct. SEM (standard error of measurement) was chosen for the stopping rule because it offers a flexible framework for modeling relationships between observed data (test items) and latent traits (ability levels), evaluating measurement precision, and enhancing testing efficiency (Sideridis et al., 2018). During the assessment process, the stopping rule can be applied to end the test when SEM falls below a specified threshold, indicating sufficient precision:

The formula 𝑆𝐸𝑀 = $\sqrt{1-Rel}=$ ⇔ 𝑅𝑒𝑙 = 1 − 𝑆𝐸𝑀^2^ allows for calculating standard measurement errors corresponding to reliabilities of 0.75, 0.80, 0.85, and 0.90, resulting in SEM values of 0.500, 0.447, 0.387, and 0.316, respectively. When the stopping rule SEM < 0.316 is applied, the procedure maintains a minimum reliability of 0.90 for the estimates of personal abilities, with the flexibility to specify different minimum reliabilities for each dimension (Bjorner et al., 2007; Chalmers, 2016). Using this approach, SEM ensures that the adaptive assessment is accurate and efficient, balancing the number of test items with the need for reliable measurement of a child’s ability. Simulation studies were conducted to evaluate the performance of the CAT algorithm, generating response patterns from simulated test subjects with fixed parameters (Magis and Raîche, 2012). The ability of IRT models to fit depends on the match between the items and the sample (skewed items require larger sample sizes, such as 500–1,000), with larger sample sizes providing better results. Given our smaller sample, we repeated parameter estimates multiple times to enhance their stability. We also conducted simulation studies to evaluate item functionality, establishing factor models based on the intended content during the data generation. The EuLeApp© was built on measuring information on the interrelationships among various early literacy dimensions. MIRT models can estimate skills with the categorical factor structure of early literacy components (Ackerman et al., 2003). Thus, the analysis process was carried out with multidimensional CAT, which is based on Multidimensional IRT (MIRT) models and allows the simultaneous measurement of more than one dimension (Segall, 2009).

## 3 Results

### 3.1 Multidimensional IRT model comparisons

A confirmatory IRT model was developed by assigning the items to six latent dimensions based on intended measurement purposes: concepts of print (Items 1–40), print awareness (Items 41–59), word awareness (Items 60–71), phonological awareness (Items 71–100), alphabet knowledge (Items 101–146), and early reading (Items 147–183).

Next, a covariance matrix was defined for a model with correlated dimensions. The number of parameters was 408 for the M2PL model, 585 for the M3PL model, and 762 for the M4PL model (Table 3). While statistical model fit is important, it should not be the only criterion for model selection; theoretical assumptions about the underlying model should also be considered (Robitzsch, 2022). Since we can theoretically substantiate that the IRT model has four parameters, we compared M2PL, M3PL and M4PL models. However, given the relatively small sample size (n = 307), it was anticipated that the model estimates of the M4PL models might lack stability (Wolf et al., 2013). When parameter estimates are unstable, this suggests the possibility of alternative models with improved parameter estimations (Robitzsch, 2022). Therefore, the M2PL, M3PL, and M4PL models were estimated multiple times using the same item allocations to further address stability concerns (e.g., the M4PL model was estimated 17 times).

**Table 3:** Model comparison in search of the optimal structure of EuLeApp©.

							Confidence interval					Items in MCAT SEM < 0.447			
Model	Nr.	Number of parameters	*M_2_*	*dF*	*p*	RMSEA	5% LB	95% UB	SRMSR	TLI	CFI	*M*	*Mdn*	*SD*	%
M2PL	1	408	16495.4	15,345	0.000	0.0157	0.0134	0.0176	0.0641	0.9929	0.9930	93.4	77	44.9	19
	2	408	16520.7	15,345	0.000	0.0158	0.0136	0.0178	0.0641	0.9928	0.9929	92.8	76	45.2	19
	3	408	16529.9	15,345	0.000	0.0159	0.0137	0.0178	0.0641	0.9927	0.9928	92.2	76	44.4	18
	4	408	16549.0	15,345	0.000	0.0160	0.0138	0.0180	0.0640	0.9926	0.9927	92.8	76	45.0	19
M3PL	1	585	16352.0	15,168	0.000	0.0160	0.0137	0.0179	0.0679	0.9926	0.9928	71.5	36	62.9	25
	2	585	16454.5	15,168	0.000	0.0166	0.0145	0.0185	0.0667	0.9920	0.9922	67.5	36	59.7	21
	3	585	16792.0	15,168	0.000	0.0187	0.0168	0.0204	0.0614	0.9899	0.9902	86.2	48	66.2	33
	4	585	16946.4	15,168	0.000	0.0196	0.0177	0.0212	0.0594	0.9889	0.9892	72.4	34	63.9	25
M4PL	1	762	15902.6	14,991	0.000	0.0141	0.0116	0.0163	0.0559	0.9943	0.9945	62.6	28	63.9	23
	2	762	16000.5	14,991	0.000	0.0148	0.0124	0.0169	0.0609	0.9936	0.9939	55.3	26	58.5	18
	3	762	16086.4	14,991	0.000	0.0155	0.0131	0.0175	0.0690	0.9931	0.9934	48.3	25	55.7	15
	4	762	16106.5	14,991	0.000	0.0156	0.0133	0.0176	0.0677	0.9930	0.9932	83.7	34	71.1	36
	5	762	16169.9	14,991	0.000	0.0160	0.0138	0.0180	0.0578	0.9926	0.9929	91.6	51	73.7	42
	6	762	16215.1	14,991	0.000	0.0163	0.0141	0.0183	0.0600	0.9923	0.9926	50.4	26	55.8	16
	7	762	16249.1	14,991	0.000	0.0166	0.0144	0.0185	0.0594	0.9921	0.9924	78.5	40	64.7	29
	8	762	16255.9	14,991	0.000	0.0166	0.0145	0.0185	0.0615	0.9920	0.9923	50.6	23	59.6	18
	9	762	16394.1	14,991	0.000	0.0175	0.0154	0.0193	0.0595	0.9912	0.9915	67.1	29	66.4	26
	10	762	16515.6	14,991	0.000	0.0182	0.0163	0.0200	0.0563	0.9904	0.9908	36.5	21	45.4	9
	11	762	16541.2	14,991	0.000	0.0184	0.0164	0.0201	0.0759	0.9902	0.9906	63.9	30	63.8	23
	12	762	16544.2	14,991	0.000	0.0184	0.0164	0.0202	0.0690	0.9902	0.9906	47.8	20	59.8	17
	13	762	16594.4	14,991	0.000	0.0187	0.0168	0.0204	0.0694	0.9899	0.9903	47.8	24	55.8	15
	14	762	16659.3	14,991	0.000	0.0191	0.0172	0.0208	0.0626	0.9895	0.9899	53.4	28	56.0	15
	15	762	16672.3	14,991	0.000	0.0191	0.0173	0.0208	0.0786	0.9894	0.9898	32.0	20	40.9	7
	16	762	16676.2	14,991	0.000	0.0192	0.0173	0.0209	0.0592	0.9894	0.9898	72.9	36	68.2	29
	17	762	16917.5	14,991	0.000	0.0205	0.0187	0.0221	0.0796	0.9879	0.9883	32.0	20	40.9	7

*M₂, Deviance; dF, Degrees of Freedom; RMSEA, Root Mean Square Error of Approximation; 5% LB, %5 Lower Bound; 95% UB, 95% Upper Bound; SRMSR, Standardized Root Mean Square Residual; TLI & CFI, Tucker-Lewis Index & Comparative Fit Index; M, Mean Number of Items in MCAT; Mdn, Median Number of Items in MCAT; SD, Standard Deviation; %, Percentage of individuals required to answer all 177 items.*

Table 3 presents the model fit indices for the M2PL, M3PL, and M4PL models, with all estimates using the final item assignments.

The results consistently indicated that M4PL models provided a superior data fit compared to M2PL and M3PL models. Specifically, M4PL models No. 1 and 2 demonstrated superior RMSEA values, while models No. 1 and 10 stood out in terms of their SRMSR values. Based on the literature by Maydeu-Olivares (2013) to prioritize SRMSR as a model fit criterion, M4PL model No. 10 was ultimately selected. Additionally, the performance of model No. 10 in the multidimensional CAT framework played a decisive role in its selection. The favored M4PL model is superior to the M2PL and M3PL models not only in terms of fit indices, but also in terms of the number of answered items required to achieve a reliability of 0.80 on the MCAT. This iterative estimation process was essential for ensuring parameter stability and validating the reliability of the adaptive testing framework, reinforcing the robustness of the final model.

Table 3 shows that M4PL-Model No. 10, based on the M4PL model, demonstrates a very strong model fit, as indicated by the following fit indices: RMSEA = 0.0182 [0.0163; 0.0200], which suggests a close fit to the data; SRMR = 0.0563, indicating a small standardized residual; TLI = 0.9904, and CFI = 0.9908, both of which suggest an excellent fit to the model.

These values collectively confirm that the model fits the observed data well, providing reliable estimates.

### 3.2 MCAT calibration of the item pool

**Figure 4:**
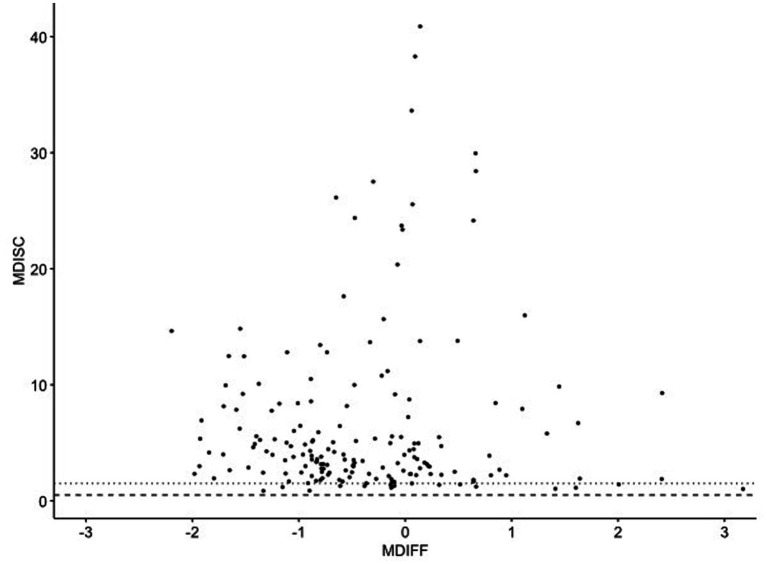
Item difficulties in the calibrated item bank. *MDISC*: Item Discrimination Index, *MDIFF*: Item Difficulty Index. The *MDIFF* values were multiplied by −1 so that the difficulty of the items increases from left to right in the diagram.

An optimally calibrated item pool for CAT should include a broad distribution of items covering the difficulty parameter range $\Delta_{j}$ , from −2 to +2. This range ensures that the item pool can assess abilities across a wide spectrum of test-takers. In addition to a well-distributed difficulty range, the discrimination parameter $A_{j}$ , plays a critical role in item selection. Higher values of $A_{j}$ indicate items that are more effective in distinguishing between individuals of different ability levels, thus contributing to the overall reliability of the test. Figure 4 presents the distribution of the difficulty parameters $\Delta_{j}$ (*MDIFF* on the x-axis) and the discrimination parameters $A_{j}$ (*MDISC* on the y-axis) for the 177 items in the item bank.

Regarding discrimination $A_{j}$ , items in the calibrated bank exhibit varying levels of effectiveness in distinguishing between children with different abilities. The majority of the items falls within the range of high discriminatory power ( $A_{j}$ > 1.5, dashed line), indicating that they are very effective in distinguishing between different levels of ability. Only a small subset of 16 items shows an acceptable discriminatory power of 0.5 < $A_{j}$ < 1.5 (area between the dotted and dashed line). In the area of average abilities (−1 < Δj < 1), there is a subset of 15 items that stand out with an excellent selectivity of $A_{j}$ >15. These items are especially valuable in increasing the precision of the ability estimates for individuals whose abilities lie near the population mean. The calibrated item pool reveals that items with the highest discriminatory power are concentrated in the range of average abilities, specifically between −1 and +1 on the ability scale. The item bank is concentrated with highly discriminating items from below-average to average abilities (−2 to +1), ensuring accurate assessment in this range. A dozen items adequately cover the above-average range (+1 to +3). This distribution ensures the test maintains precision and reliability across a broad spectrum of abilities.

#### 3.2.1 Results from the simulation study

Given the developmental range of the target sample (ages 4 to 7), it was important to examine whether differential item functioning occurs as a result of age. To determine optimal starting items, easy items were selected based on average items and relevant literature per scale for each of the three age groups (4 years and younger, 5 years, and 6 years and older). Approximate Posterior (AP) rule for item selection and the Maximum *A Posteriori* (MAP) method for estimating a person’s ability. Each age group was assigned a single starting item, resulting in a total of three starting items. The simulation was conducted twice, applying different stopping rules: SEM < 0.447 for the first and SEM < 0.387 for the second. The MCAT simulation with 307 complete cases from the training sample provided standard errors of measurement (SEM) for the estimates of the six early literacy components. The sum of squared SEM values was used as the target variable to be minimized. Then, a linear regression analysis was conducted to assess the influence of the two variables: starting item and stop rule. Table 4 shows that age-appropriate starting items significantly improved the ability to estimate precision, with significant reductions in the sum of squared errors per age group: for four-year-olds by 6.92 (−1.07 per dimension) (*t* = −3.52, *p* < 0.001), for 5-year-olds by 11.05 (−1.36 per dimension) (*t* = −5.00, *p* < 0.001), and for 6-year-olds by 10.83 (−1.34 per dimension) (*t* = −4.03, *p* < 0.001). These findings align with psychometric principles, which emphasize the importance of accounting in assessments covering a broad age range (Best and Miller, 2010; Snow, 2020).

**Table 4:** Regression analysis for identifying optimal starting items by age group.

Age groups	Items	*b*	*SE*	*t*	*p*	β
	(Intercept)	57.91	1.74	33.20	0.000	0.164
	SEM < 0.447	6.19	0.64	9.68	0.000	0.257
4;0–4;11	SK01_25boot	−6.92	1.96	−3.52	0.000	−0.288
	SK06_05mo	−3.23	2.42	−1.33	0.184	−0.134
	SK06_01buchstaben1s	−2.93	2.42	−1.21	0.226	−0.122
	SK05_27am	−1.85	2.42	−0.76	0.445	−0.077
	SK04_16e	−0.91	2.42	−0.38	0.708	−0.038
	SK01_01einbahnstraße	−0.55	2.42	−0.23	0.820	−0.023
	SK02_09buchstabe1	−0.48	2.10	−0.23	0.819	−0.020
5;0–5;11	SK05_28im	−11.05	2.21	−5.00	0.000	−0.459
	SK05_12r	−9.08	2.21	−4.11	0.000	−0.378
	SK03_06luecke	−8.50	2.21	−3.85	0.000	−0.353
	SK01_03friseur	−8.29	2.21	−3.75	0.000	−0.345
	SK05_04u	−8.11	2.21	−3.67	0.000	−0.337
	SK06_06am	−8.02	2.21	−3.63	0.000	−0.333
	SK04_15f	−7.40	2.21	−3.35	0.001	−0.308
	SK06_01buchstaben1i	−7.24	2.21	−3.28	0.001	−0.301
6;0–6;11	SK05_29po	−10.83	2.69	−4.03	0.000	−0.450
	SK05_19y	−10.18	2.69	−3.79	0.000	−0.423
	SK02_07seite1	−9.35	2.69	−3.48	0.001	−0.389
	SK04_29kuh2	−9.00	2.69	−3.35	0.001	−0.374
	SK06_04buchstaben4q	−8.97	2.69	−3.34	0.001	−0.373
	SK06_08pa	−8.72	2.69	−3.25	0.001	−0.363
	SK06_02buchstaben2r	−6.79	2.69	−2.53	0.011	−0.282
	SK03_11woerteranzahl3	−6.65	2.69	−2.47	0.013	−0.276

SK01, concepts of prints; SK02, print awareness; SK03, word awareness; SK04, phonological awareness; SK05, alphabet knowledge; SK06, early reading; *b*, Regression Coefficient; *SE*, Standard Error; *β*, Standardized Coefficient.

The reference group for the dummy-coded regression analysis is the start item SK05_05i, with a stop rule of SEM < 0.387. Since R^2^ is not meaningful in a dummy-coded regression analysis, it is not reported in this table.

Building on this precision improvement, the next phase focused on determining the most effective item selection method through systematic simulation studies comparing parameter estimates using the 4PL model in mirtCAT (Chalmers, 2016) across different item selection rules.

IRT-based CATs employ various rules to estimate children’s abilities (Yao, 2013). During the assessment process, each response continuously refines the ability estimate, allowing the system to adjust dynamically. The algorithm selects the most appropriate items to optimize ability estimation. In addition, the system adapts based on whether a response is correct or incorrect, adjusting the difficulty level of subsequent items accordingly. Typically, a correct response leads to the selection of a more difficult item, while an incorrect response results in an easier one (Ebenbeck and Gebhardt, 2024). Table 5 summarizes the results from simulation studies comparing different item selection methods (D-rule, T-rule, A-rule, W-rule, E-rule, TP-rule, AP-rule, WP-rule, and EP-rule) using predetermined start items and applying two different stop-rules: SEM < 0.447 and SEM < 0.387.

**Table 5:** Simulation studies with different item selection methods.

Stop rule	Method	D-rule	T-rule	A-rule	W-rule	E-rule	TP-rule	AP-rule	WP-rule	EP-rule
SEM < 0.447	*M*	94.7	54.1	127.5	55.1	89.3	54.1	36.5	55.1	47.4
	*SD*	37.4	50.5	40.4	50.8	55.2	50.5	45.4	50.8	54.7
	Min	71	10	31	11	20	10	9	11	10
	Median	79	31	138	32	70	31	21	32	25
	75. Per.	83	66	157	62	139	66	29	62	35
	80. Per.	86	81	161	74	144	81	31	74	42
	85. Per.	177	110	177	111	177	110	37	111	81
	90. Per.	177	177	177	177	177	177	54	177	177
	95. Per.	177	177	177	177	177	177	177	177	177
	Max	177	177	177	177	177	177	177	177	177
	Total 177 items	17%	11%	18%	12%	16%	11%	9%	12%	15%
SEM < 0.387	*M*	112.1	86.6	144.9	87.9	117.7	86.6	60.4	87.9	77.3
	*SD*	46.7	64.8	36.4	64.2	54.4	64.8	62.3	64.2	67.4
	Min.	71	14	31	15	23	14	9	15	12
	Median	81	56	154	56	132	56	29	56	40
	75. Per	177	177	177	177	177	177	57	177	177
	80. Per	177	177	177	177	177	177	177	177	177
	85. Per	177	177	177	177	177	177	177	177	177
	90. Per	177	177	177	177	177	177	177	177	177
	95. Per	177	177	177	177	177	177	177	177	177
	Max	177	177	177	177	177	177	177	177	177
	Total 177 items	34%	30%	39%	30%	34%	30%	21%	30%	31%

SEM, Standard Error of Measurement; M, Mean; SD, Standard Deviation; D-rule, Discrimination Rule; T-rule, Theta Rule; A-rule, A-optimality Rule; W-rule, Weighted Information Rule; E-rule, Entropy Rule; TP-rule, Targeting Precision Rule; WP-rule, Weighted Posterior Rule; EP-rule, Expected Posterior Rule.

When applying the stop-rule SEM < 0.447, which corresponds to a minimum reliability of 0.80, the analysis revealed that a minimum of 9 items was required, with a mean of 36.5 items and a median of 21 items for the calibration sample; notably, 90% of the tests were completed within 54 items. With a stricter stopping criterion SEM < 0.387 (reliability = 0.85), the AP rule again performed best, resulting in 75% of tests being completed within 57 items. The AP-rule showed the most optimal item selection method, requiring fewer items to achieve the desired level of precision, as demonstrated by both stopping rules (SEM < 0.447 and SEM < 0.387). Based on these findings, the following stop-rule strategy was developed: (a) the first 49 items are evaluated using SEM < 0.387, ensuring that approximately 75% of cases are tested with a minimum reliability of 0.85. From the 50th item, the stop rule switches to SEM < 0.447, covering an additional 15% of cases with a minimum reliability of 0.80. Beyond the 60th item, the remaining 10% of cases—those requiring all 177 items—are addressed. In these cases, the procedure calculates the sum of squares of the standard error ranges for the six dimensions over the last 10 items, terminating when the sum falls below 0.0005. An overview of the stop rules applied across the six Early Literacy dimensions is presented below:

- Stop rule for items 1–49: SEM < 0.387298334620742
- Stop rule from item 50 onwards: SEM < 0.447213595499958
- Additional stop rule from item 60: $\overset{6}{\underset{f=1}{\sum }}{\left(SEM_{f}^{\text{max}}-SEM_{f}^{\text{min}}\right)}^{2}<0.0005$

If the threshold of 0.0005 is met, it indicates that the standard errors of measurement over the last 10 items differ by less than $\sqrt{0.0005/6}=$ 0.01 per dimension. This shows that the standard errors are stable, and cannot be significantly reduced by additional items. According to the stopping rules developed using the calibrated data (Table 6), 75% of children are tested with up to 50 items at a minimum reliability of 0.85, while the remaining cases are tested with up to approximately 75 items at a minimum reliability of 0.80.

**Table 6:** Results of MCAT simulations for the number of items per test and the reliability obtained for each dimension.

Sample		Items	Rel. CP	Rel. PAW	Rel. WA	Rel. PA	Rel. AK	Rel. FR
*N* = 307	Mean	35.0	0.897	0.929	0.944	0.920	0.939	0.869
	*SD*	18.8	0.064	0.051	0.037	0.045	0.059	0.065
	Min	9	0.558	0.619	0.682	0.659	0.578	0.522
	5. Per.	15	0.804	0.862	0.899	0.855	0.804	0.751
	25. Per.	21	0.863	0.899	0.925	0.894	0.938	0.853
	50. Per.	29	0.913	0.928	0.945	0.924	0.960	0.864
	75. Per.	50	0.942	0.973	0.973	0.958	0.973	0.893
	95. Per.	73	0.965	0.988	0.984	0.974	0.981	0.980
	Max	118	0.979	0.989	0.989	0.982	0.993	0.989

According to the stopping rules developed with the calibrated data, 75% of cases are tested with up to 50 items at a minimum reliability of 0.85, and the remainder are tested with up to approximately 75 items at a minimum reliability of 0.80. For the EuLeApp© dimensions of concepts of print (CP), print awareness (PAW), word awareness (WA), phonological awareness (PA), alphabet knowledge (AK), and first reading (FR), the actual reliabilities are between 0.804 (5th percentile) and 0.988 (95th percentile). It is noticeable that the reliability of the first EuLeApp© dimension of concepts of prints does not reach the reliability of the other five dimensions.

**Figure 5:**
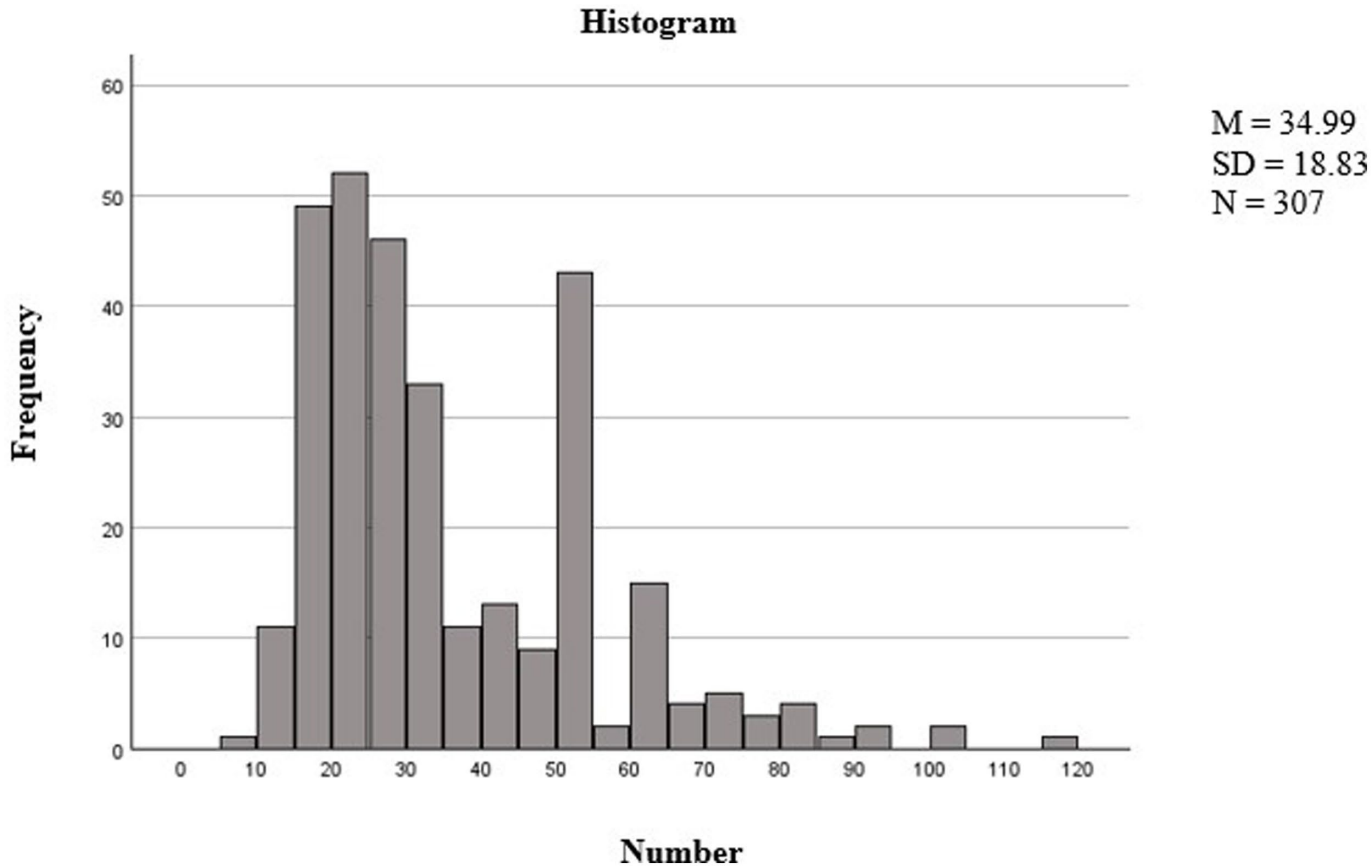
Distribution of item selection per test based on stop rule. The figure shows the distribution of the number of items completed (x-axis labeled “number of items”) and their frequency (y-axis labeled “frequency”).

Figure 5 shows the effectiveness of the stop rules and item selection methods, where most participants did not need to complete the maximum number of items. Accordingly, in the distribution of the number of items per early literacy scale, it is evident that tests with up to 50 items form a distinct population.

The two minor peaks observed at around 50 and 60 items correspond to the application of stop rules, such as SEM < 0.447 or SEM < 0.387, which marks the end of the test for many participants at these points. Items that show adequate fit to a particular IRT model can be assumed to tap into the construct as specified by the model (Chan et al., 2015), while items that show poor fit may measure a different dimension that is not captured by the model specified.

**Figure 6:**
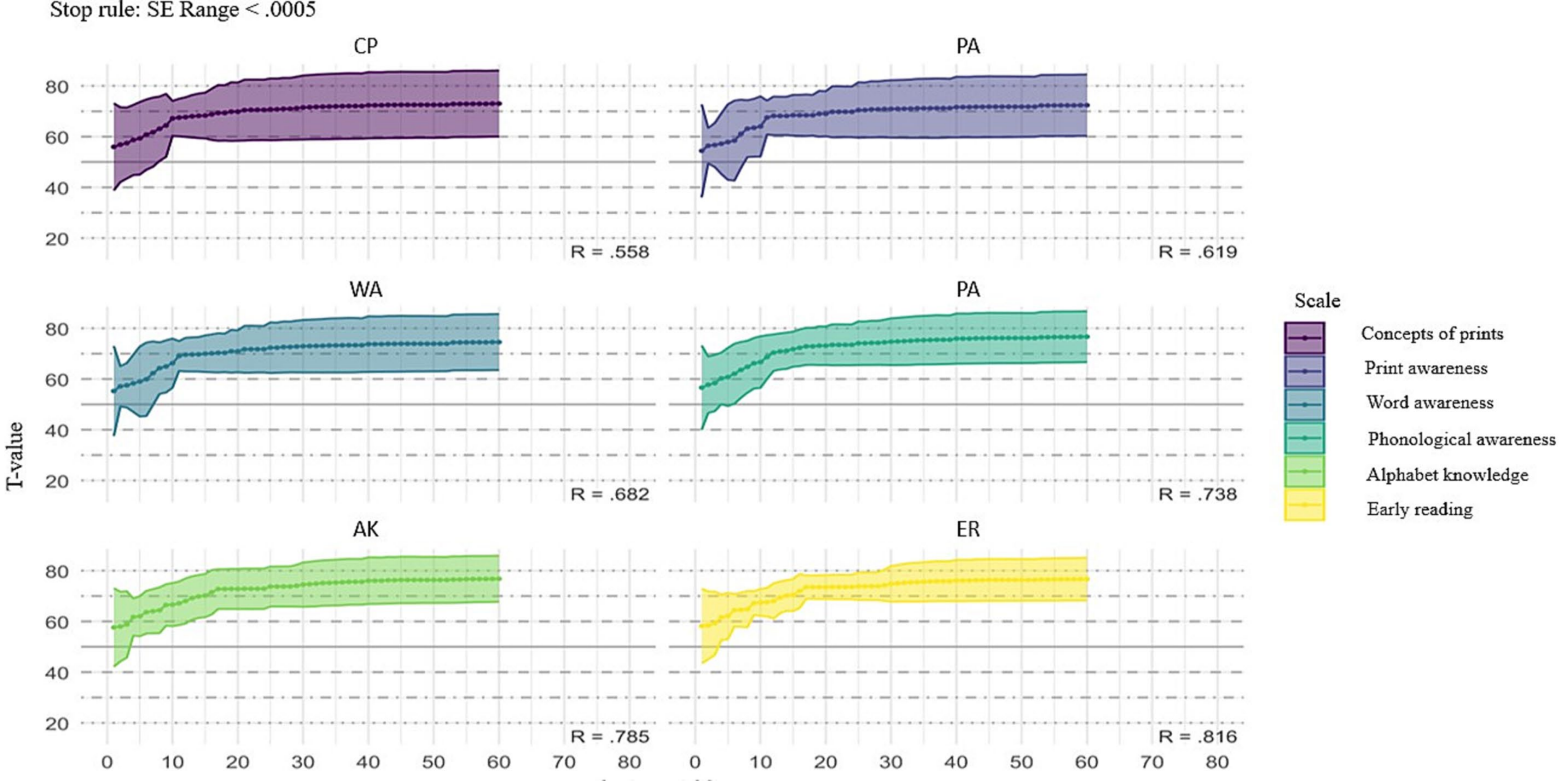
Test of *t*-scores across six scales using stop rule when standard errors (SE) < 0.0005. The solid lines represent the average *t*-scores, while the shaded areas indicate the standard error ranges.

The stop-rule effectively controls standard error, allowing for reliable ability estimates around the 50th item in most cases, as shown in Figure 6. The dimensions display varying convergence rates in T-scores as more items are answered, with most dimensions stabilizing after approximately 50 to 70 items. For example, dimension SB shows slower T-score stabilization with an R-value of 0.558, indicating moderate reliability. However, dimension EL stabilizes more quickly and shows higher reliability with an R-value of 0.816, as evidenced by the narrower confidence bands and consistent T-scores earlier in the item sequence. Similarly, BK demonstrates strong reliability, with an R-value of 0.785.

### 3.3 Test–retest reliability

The stability of all measures over time was examined through a correlation analysis. T1 took place in Fall 2023, followed by T2 in Spring 2024. As shown in Table 7, which presents both within-time correlations and T1-T2 test–retest correlations, each measure demonstrated good reliability across the two time points. The strong correlations (*p* < 0.01) among literacy components suggest that these skills are highly interrelated, with each influencing or aligning closely with the others.

**Table 7:** Combined within-time & test–retest correlations of early literacy skills.

				Pearson correlations						
Variable	*n*	*M*	*SD*	1	2	3	4	5	6	T1-T2
T1										
Concept of prints	307	23.91	10.41	–						0.76*
Print awareness	307	12.12	3.86	0.61*	–					0.71*
Word awareness	307	5.36	3.20	0.61*	0.68*	–				0.73*
Phonologic awareness	307	18.91	4.27	0.51*	0.53*	0.55*	–			0.64*
Alphabet knowledge	307	19.57	9.14	0.53*	0.45*	0.48*	0.57*	–		0.88*
Early reading	307	7.14	9.05	0.49*	0.39*	0.40*	0.53*	0.86*	–	0.77*
T2										
Concept of prints	159	28.26	9.68	–						–
Print awareness	159	13.77	3.56	0.64*	–					–
Word awareness	159	6.89	3.33	0.66*	0.67*	–				–
Phonologic awareness	159	20.77	4.45	0.56*	0.56*	0.59*	–			–
Alphabet knowledge	159	21.27	8.61	0.57*	0.49*	0.61*	0.60*	–		–
Early reading	159	8.61	8.86	0.50*	0.42*	0.56*	0.53*	0.84*	–	–

*p < 0.01.

## 4 Discussion

This study aimed to develop a conceptual framework for adaptive early literacy assessment tools using MCAT based on Item-Response Theory (IRT). Our findings will be discussed concerning two key points: the psychometric properties of the tablet-based assessment tool and its applications in early literacy education.

The results highlight the powerful psychometric properties of the EuLeApp©, which employs a Multidimensional Computerized Adaptive Testing (MCAT) methodology. The current distribution of item completion demonstrates the effectiveness of this adaptive mechanism (Keuning and Verhoeven, 2008), with most children completing the assessment after a variable number of items, reflecting its tailored nature. This approach ensures that the assessment dynamically adjusts to each child’s ability level, thereby improving the efficiency and precision of early literacy skill measurement. Simulation studies further indicate that the proposed method optimizes item selection, enhancing the accuracy of ability estimates by leveraging the CAT framework, which adjusts item difficulty based on a child’s performance. This adaptive approach yields a more individualized assessment, offering greater precision in measuring early literacy skills compared to traditional fixed-length tests. The method also considers test-taking participation in item selection, which is especially beneficial for children with lower ability levels, particularly in low-category (CAT) settings (Gorgun and Bulut, 2023). In sum, these findings have significant implications for measurement practices in adaptive testing (Weiss, 2004; Yen et al., 2012).

Additionally, while computerized adaptive testing (CAT) typically operates more effectively with simpler models such as the 1PL and 2PL, applying the 4PL model in this study presents areas that warrant further exploration. In this study, the 4PL-IRT model gave better results when using item parameters as an item pool and estimating children’s ability levels based on the entire test (183 items). As we mentioned before, one reason for this may be that children’s attention may decrease after the 10th minute of the test, which lasts approximately 20 min. A parameter that accounts for the probability of carelessness, modeling the chance of a correct answer even when the test child has, the 4PL model could better capture situations where children with sufficient ability to answer questions correctly fail due to a lack of attention or motivation. While the 4-Parameter Logistic (4PL) Model offers flexibility by accounting for guessing and the possibility of a high-performing test child not answering correctly, it may show a weak estimation of item difficulty or discrimination used for adaptive testing with Item Response Theory (IRT). However, to mitigate these challenges, many models have been tested in the calibration phase of the item parameters to ensure that the lower and upper asymptotes are estimated accurately and the items are carefully pre-calibrated. In addition, restrictions were applied to the parameter ranges or regularization techniques of the 4PL model to prevent overfitting and enhance the robustness of the parameter estimates. While these methods helped mitigate some potential issues, further research is necessary to clarify these adjustments’ impact more clearly. Specifically, future studies should explore how these restrictions influence the accuracy and stability of parameter estimates within the 4PL model, particularly in early literacy assessment contexts. Investigating the long-term effects of using the 4PL model across different populations and educational settings will provide greater insight into its practical value. Continued evaluation and iterative development of EuleApp© will allow for a more robust tool that meets the needs of both educators and young learners better, supporting more precise assessments of early literacy skills.

The EuLeApp© assessment tool has the potential to effectively identify children’s strengths and areas of weaknesses in early literacy development. Specifically, its adaptive, data-driven, and multidimensional framework may offer advantages over traditional methods in educational settings. The findings indicate that EuLeApp© can tailor assessments to each child’s individual ability levels through sophisticated item selection rules. This dynamic adjustment enables more precise and targeted literacy evaluations than traditional assessments, which typically do not modify item difficulty in response to a child’s performance during testing. Moreover, the present study has some important implications for screening children early for possible early reading-writing problems and dyslexia. The literature documents the relationship between early literacy skills and reading achievement well (Whitehurst and Lonigan, 1998; Justice et al., 2009; Jiménez et al., 2024) and highlights the potential for early monitoring of these skills (Catts et al., 2001; Neumann and Neumann, 2014). Additionally, other research has pointed out that early comprehensive and accurate assessments provide stronger predictions regarding dyslexia risk and later reading problems (Catts et al., 2001; Catts et al., 2015). However, studies have often been limited by digital assessment tools that focus only on one or two early literacy domains (e.g., Golinkoff et al., 2017; Jonathan Castañeda-Fernández et al., 2023; Neumann, 2018). Our findings showed that our more comprehensive screening app can identify children at risk for early literacy, which is crucial for later reading comprehension and school success, with acceptable level of accuracy. The potential of the EuleApp© to accurately measure literacy skills suggests that it could serve to identify children who may benefit from early interventions, thus mitigating the risks associated with dyslexia and other literacy challenges. It is important to note that children enter kindergarten with varying levels of language and cognitive skills, which are critical predictors of future reading success. In the standardization process of this assessment tool, our initial focus was on evaluating children with typical language development to establish baseline performance measures. In the next phase, further development of this screening tool is needed to ensure that children’s early literacy accurately distinguishes among diverse children. Overall, using the tool in this way in an early educational setting would be useful for both identifying literacy practices in everyday practice integration, enabling teachers to reflect on their practice, and monitoring progress.

In conclusion, the integration of adaptive, data-driven tools like the EuleApp© in educational settings not only addresses the need for precise assessments but also underscores the critical importance of proactive measures in supporting children’s literacy development. The results showed that the individual component scores exhibit adequate psychometric properties, including sufficient precision and validity, supporting the reliability of the assessment tool in measuring early literacy skills effectively.

## 5 Limitations

This study presents several limitations that should be acknowledged. One limitation of this research is the relatively small sample size. A larger number of participants would facilitate more precise calibration of item parameters and enhance the accuracy of ability estimates across diverse groups (Bjorner et al., 2007). Although the current sample size was sufficient to conduct the Computerized Adaptive Testing (CAT) analysis, employing a larger sample would improve the generalizability and robustness of the results. The findings of this study may also lack generalizability due to the limited diversity within the sample. This research was predominantly conducted with a German population and in the German language, which may not fully represent the experiences of children from diverse cultural, linguistic, or socioeconomic backgrounds. The composition of the sample, mainly consisting of participants from middle- and high-socioeconomic-status regions, may present a potential limitation regarding the interpretation of item parameters. In other words, due to their exposure to more literacy-rich environments, these children may have found certain items easier, potentially resulting in a ceiling effect. For instance, assessments of early phonemic awareness (Burt and Barbara Dodd, 1999), alphabet knowledge, or early reading skills (Bowey, 1995; Dolean et al., 2019) may have been less discriminatory for children with early literacy advantages.

Future studies should incorporate more varied samples by increasing the sample size and including participants from diverse cultural and socioeconomic backgrounds to enhance the current findings. Ensuring that all young children have the opportunity to develop proficiency in literacy skills is a key area of focus globally. Consequently, further research involving different populations is essential for exploring the broader applicability of the EuleApp© in various linguistic contexts and ensuring its effectiveness across diverse educational settings. In addition, the study’s design may limit the depth of insight into the effectiveness of the EuleApp© assessment tool. For instance, a cross-sectional design captures data at a single point in time, which may not adequately reflect the changes in children’s literacy skills over time. Longitudinal studies could provide more comprehensive insights into how children’s abilities develop and how effectively the tool tracks these changes.

## Data Availability

The raw data supporting the conclusions of this article will be made available by the authors, without undue reservation.
